# Editorial: Maternal obesity's impact on the mother and neonate

**DOI:** 10.3389/fped.2022.1042659

**Published:** 2022-10-05

**Authors:** Michael J. Horgan, Rubia Khalak, Asha Rijhsinghani

**Affiliations:** ^1^ Department of Pediatrics, Albany Medical College, Albany, NY, United States; ^2^ Department of Obstetrics and Gynecology, University of Washington, Seattle, WA, United States

**Keywords:** maternal obesity, maternal obesity and antenatal care, gestational diabetes, gestational hypertension, hypoxic ischaemic encehalopathy, neonatal respiratory distress, neonatal hospitalization costs

**Editorial on the Research Topic** Maternal obesity's impact on the mother and neonate By Horgan MJ, Khalak R, Rijhsinghani A. (2022) Front. Pediatr. 10: 1042659. doi: 10.3389/fped.2022.1042659

## Introduction

The continued rise in maternal obesity regardless of age or socioeconomic background has become a relentless problem worldwide. More women are obese at their first prenatal visit, then subsequently gaining more weight during pregnancy than ever before. Maternal obesity has been shown to compromise not only the health of the mother, but also that of the neonate. Pregnant women with co-morbidities who also have obesity fair a much greater risk of worsening of their underlying disease while pregnant. Maternal obesity has been noted to increase the inflammatory response of organs such as the placenta with subsequent downstream effects on the fetus. Although there is literature that currently exists on maternal complications from obesity and to some degree concerning the neonate, aspects of infant outcomes associated with maternal obesity remains limited. Long term effects of obesity on mothers and later childhood effects on their offspring are minimally seen in the literature.

The aim of this special issue of Frontiers in Neonatology Research Topic “*Maternal Obesity's Impact on the Mother and Neonate*” is to present the available knowledge of the impact of maternal obesity on the health of the mother and infant and showcase original work done in the field to address areas where a paucity of literature continues to exist.

## Overview of maternal obesity, management, and placental pathology

With the ever-increasing incidence of people with obesity, a mirrored increase in maternal obesity has also been noted, particularly in developed countries (1, 2). As more women are diagnosed with obesity, the rates of antepartum, intrapartum and postpartum complications and adverse effects on the neonate have been reported in the literature (3, 4).

Several research groups have found an association of increased incidence of gestational diabetes, gestational hypertension, and the need for Cesarean delivery (5, 6). Researchers have noted increased rates of intrapartum infection, preterm delivery, prematurity, congenital anomalies, stillbirth and severe hypoxic ischemic encephalopathy (HIE) in infants of mothers with obesity (5–9). Guidelines for the care of mothers with obesity have been recommended by the American College of Obstetrics and Gynecology (10). In this issue, Sterrett et al. provide a comprehensive overview of the considerations that should be undertaken when providing care to a pregnant woman with obesity or with super-morbid obesity. The authors describe the antepartum and intrapartum concerns of increased risks such as hypertension, diabetes, and pre-eclampsia. They also present the increased surgical risks and importance of being aware of the availability of additional supports at a particular facility prior to delivery. Also in this Frontiers issue is a review by Monaco-Brown and Lawrence of the how obesity can act as a stressor on the pregnant mother. The subsequent inflammatory and cytokine changes have downstream effects that can bring about changes in placental structure and function. These changes can result in the co-morbidities that are often associated with maternal obesity.

## Neonatal impact on infants of mothers with obesity and economic costs

Maternal morbidities secondary to obesity have been shown to have an impact on the infant. Some of these effects can manifest in both the immediate and early newborn period. Researchers have proposed that the changes in the intrauterine environment increase the risk for delayed fetal lung maturity leading to increased rates of transient tachypnea of the newborn (TTN) and respiratory distress syndrome (RDS) (11, 12). Findings of neonatal hypoglycemia have also been associated with maternal diabetes and maternal obesity (13). Kureshi et al. present in this issue their investigation of respiratory transition diagnoses and hypoglycemia in neonates born to mothers with obesity. The researchers found that infants born to mothers with obesity were more likely to have diagnoses of TTN, RDS and hypoglycemia in the early newborn period.

Villamor et al. found that there was an increased risk of neonatal complication of asphyxia or HIE with lower Apgar scores seen in neonates born to mothers with diabetes and obesity (14). A brief report from our center showed an association of maternal obesity and HIE (15). In this issue, Monaco-Brown et al., present their findings from a population-based cohort study using regional data. They looked at whether infants born to mothers with obesity were at higher risk of developing HIE. In their analyses of over 97,000 pregnancies, they found that infants born to mothers with obesity were more likely to have a lower 5-minute Apgar score and more likely to receive the diagnosis of HIE. An increased treatment with therapeutic hypothermia in these infants was also noted.

Despite research on maternal obesity and the impact on the neonate, the effect on healthcare costs has had minimal study. Kim et al. showed in a meta-analysis review that obesity and its' comorbidities attributed to almost one third of the medical costs of the hospitalized patient with obesity (16). From a data set of Florida's healthcare charge costs, Whiteman et al. in a 2015 study concluded that infants born to mothers with obesity had higher in-patient hospital costs when compared to infants of non-obese mothers (17). Presented here, Azher et al. assessed whether hospital care costs were greater for mothers with obesity, and their infants during the neonatal period. The authors found an increase in healthcare costs, and greater length of stays for both mothers with obesity and their infants.

## Conclusion

The compilation of articles in this Frontiers issue broadly encompasses the available literature on the topic of maternal obesity and its impact on the neonate. We hope that readers will have a sense of not only what has been studied but also be able to identify areas of future study such as the long-term effects of maternal obesity on both the mother and child.
